# Supplementary material for: Eco-Friendly Electroless Template Synthesis of Cu-Based Composite Track-Etched Membranes for Sorption Removal of Lead(II) Ions
Source: Membranes (Basel). 2023 May 7;13(5):495. doi: 10.3390/membranes13050495 (PMC10220658; doi:10.3390/membranes13050495)
Supplement: Supplementary file 1 [file membranes-13-00495-s001.zip › membranes-2361911-supplementary.pdf]

Supplementary information

Eco-friendly electroless template synthesis of Cu-based composite track-etched membranes for sorption removal of lead(II) ions

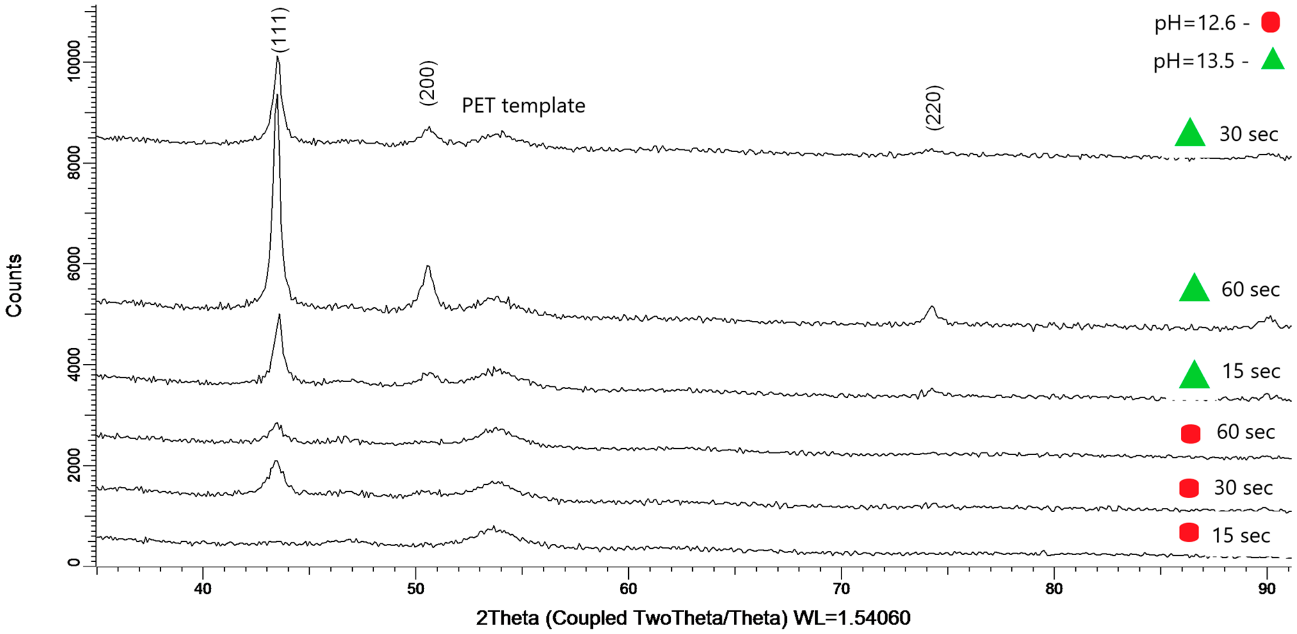

Figure S1 X-Ray diffraction (XRD) patterns of the Cu_Gly@PET composite TeMs deposited at the various deposition time

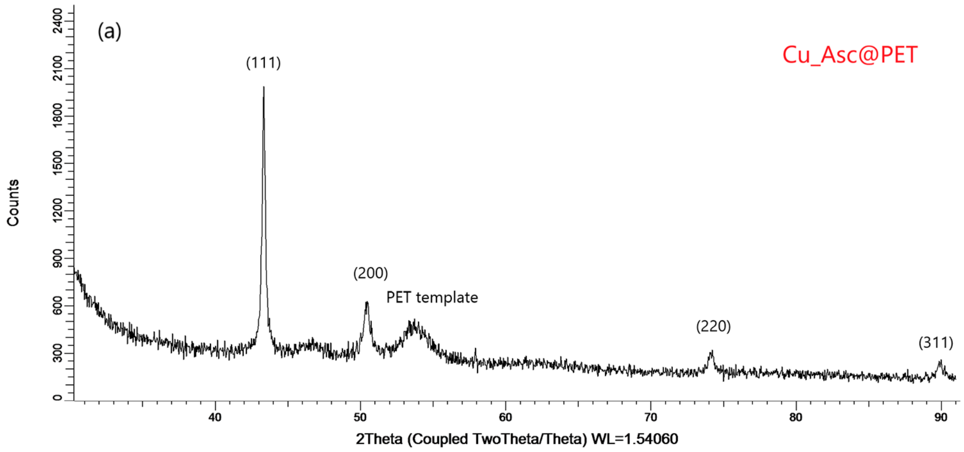

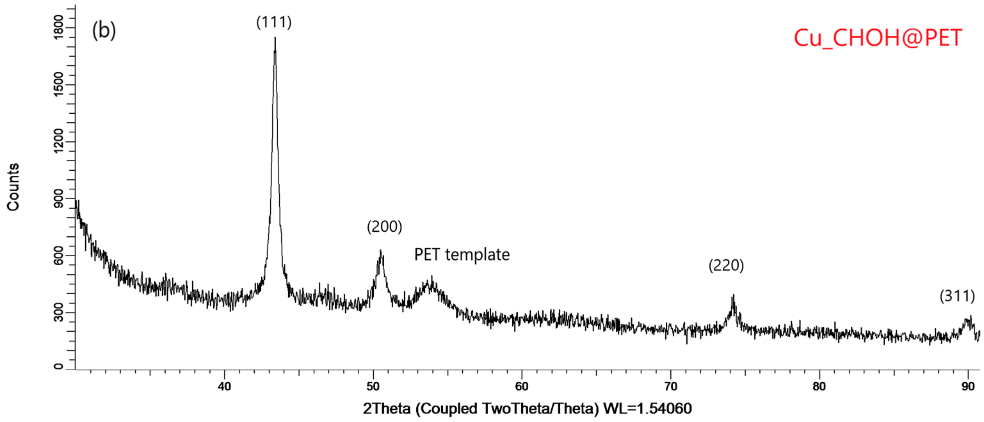

Figure S2 X-Ray diffraction (XRD) patterns of the Cu_Asc@PET (a) and Cu_CHOH@PET (b) composite TeMs

Table S1 - XRD data of composite membranes prepared with ascorbic acid and formaldehyde as a reducing agent

Composite	Phase/ content, %	(hkl) [a]	2θ°	d, Å [b]	L, nm [c]	Cell parameter, Å [e]	FWHM [d]	Volume, Å^3
Cu_Asc@PET	Cu/100	111	43.384	2.08403	38.89	a=3.608	0.244	46.97
		200	50.384	1.80968	19.92		0.490	
		220	73.999	1.27997	27.59		0.401	
		311	89.845	1.09158	27.66		0.451	
Cu_CHOH@PET	Cu/100	111	43.410	2.08284	20.36		0.466	46.88
		200	50.537	1.80457	17.37		0.562	
		220	74.139	1.27790	41.94		0.294	

[a] Miller indices for corresponding planes; [b] spacing between planes; [c] average crystallite size; [d]full-width at half-maximum; [e] crystal lattice parameter.

Table S2. Structural properties of the composite track-etched membranes (TeMs).

Composite	Structural Parameters of Embedded MTs, nm		Deposition Rate, R, mg/(cm^2 h)	Amount of Cu Loaded, mg/cm^2
	Wall Thickness	Inner Diameter		
Cu_CHOH@PET	73.7 ± 8.5	269.0 ± 10.5	5.52	0.77 ± 0.03
Cu_Asc@PET	15.2 ± 3.7	352.6 ± 12.0	0.56	0.67 ± 0.06
